# Evidence for the Induction of Anxiolytic‐Like Behavior and Memory Preservation by (+)‐Dihydrocarvone Through Studies in Adult Zebrafish and In Sílico

**DOI:** 10.1002/cbdv.71612

**Published:** 2026-08-21

**Authors:** Isadora Freitas de Sousa, Francisco Rogênio da Silva Mendes, Maria Kueirislene Amâncio Ferreira, Antônio Wlisses da Silva, Leticia Souza da Silva, Jane Eire Silva de Alencar Menezes, Márcia Machado Marinho, Emmanuel Silva Marinho, Matheus Nunes da Rocha, Antônio César Honorato Barreto, Alexandre Magno Rodrigues Teixeira, Hélcio Silva dos Santos

**Affiliations:** ^1^ Graduate Program in Natural Sciences Campus Itaperi State University of Ceará Fortaleza Ceará Brazil; ^2^ Graduate Program in Biological Chemistry Department of Biological Chemistry Regional University of Cariri Crato Ceará Brazil; ^3^ Science and Technology Centre ‐ Course of Chemistry State University Vale Do Acaraú Sobral Ceará Brazil

**Keywords:** anxiety, ketone monoterpene, memory, zebrafish

## Abstract

Anxiety and seizures are prevalent conditions worldwide, with conventional treatment relying on benzodiazepines. However, the long‐term use of these drugs is associated with adverse effects and the development of dependence, driving the search for safer therapeutic alternatives. (+)‐Dihydrocarvone is a monoterpene ketone with documented biological activities, including antifungal, antiparasitic, and anti‐inflammatory effects. This study utilized adult zebrafish (*Danio rerio*), an animal model widely used in pharmacological and behavioral research due to its neurobiological similarity to mammals. Toxicity tests, assessments of anxiolytic activity and memory effects, and *in silico* studies were conducted. The results demonstrated anxiolytic‐like effects and memory preservation associated with (+)‐dihydrocarvone, with pharmacological data suggesting the possible involvement of GABAergic and serotonergic pathways. Furthermore, molecular interaction analyses revealed affinity for the GABA∼A receptor, indicating a potential pharmacological effect.

## Graphical abstract

(+)‐Dihydrocarvone exhibited anxiolytic‐like and memory‐preserving effects in adult zebrafish. Behavioral assays showed reduced anxiety and improved cognitive performance, while in silico docking revealed strong interactions with GABAergic and cholinergic targets. These findings suggest (+)‐dihydrocarvone as a promising neuroactive compound for anxiety modulation and memory protection.

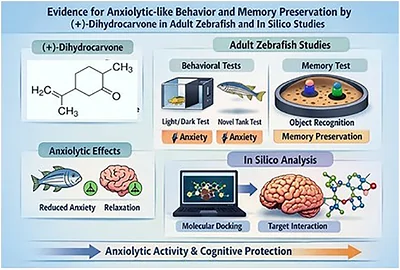

## 1 Introduction

Anxiety is a common feeling present in people's daily lives. However, when characterized by excessive anticipation of the future, intense fear, and constant worry, it can become pathological and compromise physical and mental health. Among the main symptoms cited are irritability, muscle tension, hypervigilance, restlessness, changes in sleep quality [1], and impaired cognitive functions, especially memory [2].

Seizures are neurological manifestations that affect the central nervous system (CNS), resulting from an imbalance in brain electrical activity, characterized in particular by excessive and synchronized neuronal discharges, which can cause involuntary movements, motor, sensory, or autonomic changes, as well as possible loss of consciousness [3]. These episodes may be related to other comorbidities, especially epilepsy. Studies indicate that people affected by this condition experience stressful situations that compromise their quality of life. In addition, they are more prone to developing anxiety and memory impairment [4].

The pharmacological treatment of both conditions largely involves the use of benzodiazepines, which act on the CNS by modulating GABA(_A)_receptors [5]. However, prolonged use of these drugs can cause adverse effects such as lethargy, dizziness, and irritability, as well as dependence [1]. Given these limitations, a growing search for new compounds that have anxiolytic activity with fewer side effects, aiming to promote a better quality of life and greater well‐being for the patient.

Thus, (+)‐dihydrocarvone is a monoterpene organic compound of broad economic relevance, present in various industrial sectors, such as a flavoring additive [6] or biopesticide [7]. According to the literature, this ketone monoterpene compound has biological activities that include antifungal [8], antimicrobial [9], and anti‐inflammatory [10] actions. The zebrafish (*Danio rerio)* animal model has been highlighted in the literature for its widespread use in various segments, including studies of the sensory system, digestive tract function, and neurological disorders. In addition, zebrafish have a significant advantage in behavioral investigations, which allows the observation of neural responses, favoring neuropharmacological analyses [11].

Thus, zebrafish constitute a robust animal model for in vivo studies, contributing efficiently to the preclinical evaluation of potential drug candidates. This prominence is due to the small size, ease of handling, and high reproductive rate of zebrafish. Furthermore, this alternative model has about 70% genomic similarity to humans, which significantly expands the possibilities for biological and pharmacological investigations [5] as well as metabolic similarities [12] and acts as a biomarker for toxicity studies [13].

However, the present study aims to investigate the potential anxiolytic effects of (+)‐dihydrocarvone and to evaluate its possible anticonvulsant activity in zebrafish. In addition, it seeks to perform its spectroscopic characterization, evaluate its toxicity, and analyze intermolecular interactions with CNS receptors, aiming to develop a possible therapeutic agent for the treatment of pathologies.

## 2 Results and Discussion

### 2.1 Structural Characterization (ATR‐FTIR)

**FIGURE 1:**
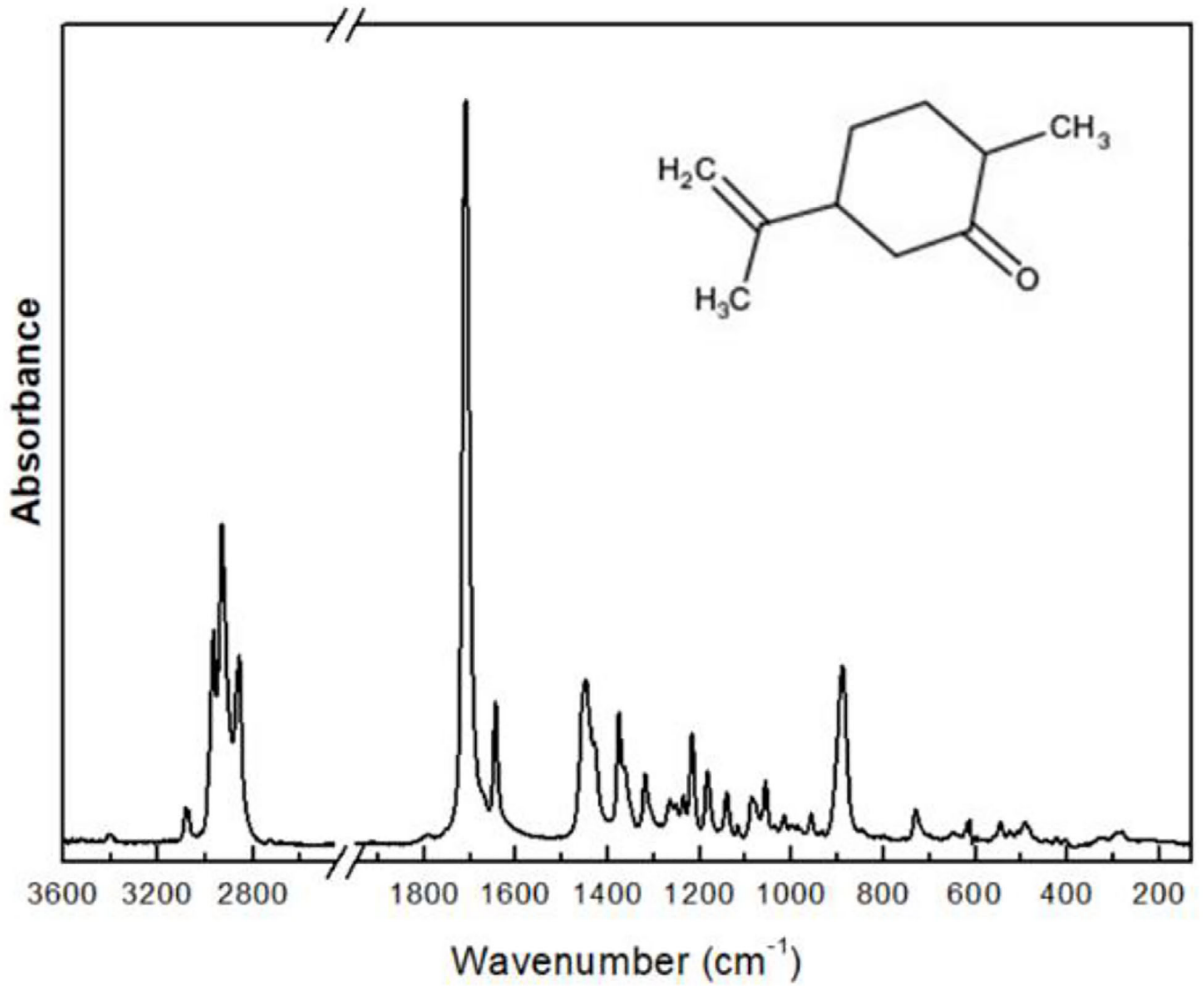
Spectroscopic analysis by ATR‐FTIR of (+)‐dihydrocarvone.

Characterization by attenuated total reflection fourier transform infrared (ATR‐FTIR), revealed the functional groups in the chemical structure of (+)‐dihydrocarvone (Figure 1), as described in Table 1.

**TABLE 1:** Main infrared absorption bands for (+)dihydrocarvone.

Wavenumber (cm^−1^)	Assignment of the vibrational modes
725	CH_3_ Bend (rocking)
1319	CH_2_ Bend (wagging)
1450	CH_3_ Bend (wagging)
1640	C═C Stretch
1701	C═O Stretch
2857	C─H Stretch
2931	C─H Stretch
3075	═C─H Stretch

The ATR‐FTIR spectrum of (+)‐dihydrocarvone shows a strong absorption corresponding to the carbonyl stretching mode at 1701 cm^−1^. Characteristic bands of bending modes of the methyl group appear at 745 and 1450 cm^−1^, which are associated with rocking and wagging vibrations, respectively. Wagging mode associated with the methylene group is found in the ATR‐FTIR at 1319 cm^−1^. The spectrum also displays a distinct C═C stretching band at 1640 cm^−1^ and various C─H stretching vibrations at 2857, 2931, and 3075 cm^−1^.

### 2.2 Acute Toxicity

The monoterpene (+)‐dihydrocarvone showed no observable toxic effects at the lower doses administered to adult zebrafish, demonstrating a favorable safety profile within the investigated dose range. Mortality was observed only at the highest dose tested (40 mg/kg), where 33.3% of the animals died (2 out of the 6 animals treated at this dose) (Table 2). Furthermore, during behavioral assays assessing anxiety, PTZ‐induced seizures, and memory—using distinct groups of animals for each test—no behavioral changes suggestive of toxicity were observed, such as marked increases or decreases in opercular movements, excessive air gulping at the surface, or episodes of erratic swimming.

**TABLE 2:** Results of acute toxicity tests for (+)‐dihydrocarvone.

Sample	Mortality				96 h LD_50_ (mg/kg) / CI
	NC	D1	D2	D3	
(+)‐dihydrocarvone	0	0	0	33.3 %	> 40

Legend: Group: (+)‐dihydrocarvone; NC, Negative control (DMSO 3%); D1, dose 1 (4 mg/kg); D2, dose 2 (20 mg/kg); D3, Dose 3 (40 mg/kg); LD(_50)_‐ lethal dose to kill 50% of adult zebrafish; CI, confidence interval.

These findings are consistent with the results of Cavalcante [14], who, when evaluating the acute toxicity of the monoterpenes R(+)‐limonene, S(‐)‐limonene, and their oxidized derivatives (alcohol, aldehyde, and perillyl acid) in zebrafish, did not observe toxicity at the same concentrations used in this study. Similarly, Todirascu–Ciornea [15] reported the absence of toxicity in zebrafish for the essential oil of Schinus terebinthifolius, whose composition is rich in monoterpenes, including limonene. Toxicological characterization is a fundamental step for the advancement of preclinical and clinical trials. By establishing safety parameters, it is possible to predict risks and monitor the pharmacological behavior of the substance in living models. Determining the relationship between the toxic dose and the therapeutic dose allows for the definition of the therapeutic index of the compound, establishing the necessary safety margin for its potential development as a drug [16].

### 2.3 Evaluation of Locomotor Activity

**FIGURE 2:**
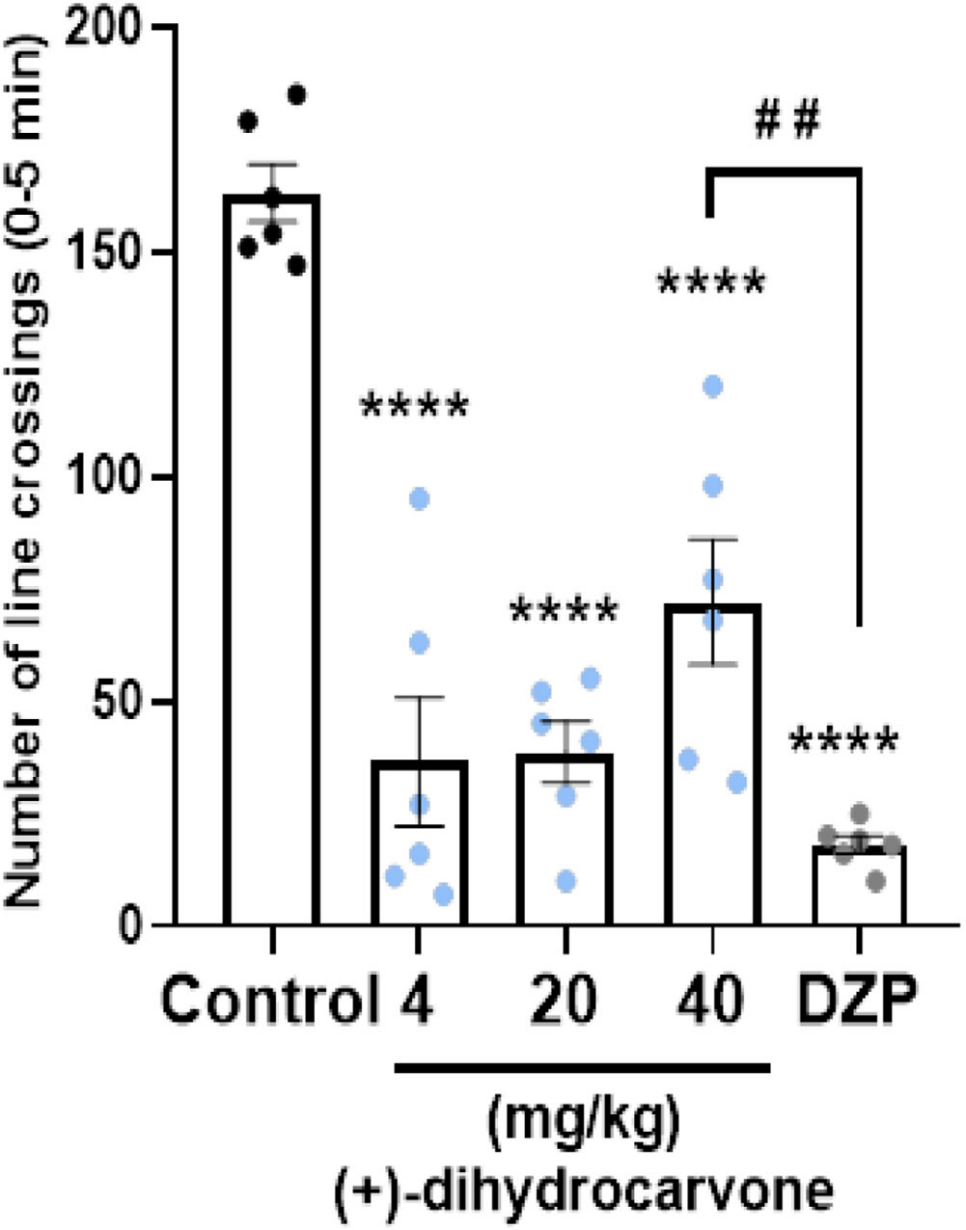
Open field test (+)‐dihydrocarvone on locomotor activity in adult zebrafish (0–5 min). A‐ (+)‐dihydrocarvone (4, 20, and 40 mg/kg). Values represent the mean ± standard error of the mean for 6 animals/group; ANOVA followed by Tukey's test. (****p < 0.0001 vs. control; ##*p* < 0.01 vs. DZP).

One‐way ANOVA analysis indicated that (+)‐dihydrocarvone induced significant locomotor changes at doses of 4, 20, and 40 mg/kg (****p < 0.0001 vs. control) compared to the control group (3% DMSO). The observed activity profile was similar to that of the group treated with diazepam (DZP, 4 mg/kg), an anxiolytic and sedative drug. The highest dose (40 mg/kg) showed a statistically significant difference regarding the effect of DZP on motor impairment (##*p* < 0.01 vs. DZP), indicating that this higher dose did not cause severe motor impairment—as was observed with diazepam—as illustrated in Figure 2. These results align with literature findings regarding other terpenes, such as the monoterpene (–)‐borneol [17] and the triterpene ursolic acid [18], which also significantly reduced swimming activity in zebrafish models.

The antidepressant potential of (R)‐(–)‐carvone was recently investigated in mouse models [19]. The results obtained in the open field test indicated that doses between 25 and 200 mg/kg altered the locomotor activity of these animals. These findings corroborate the data observed in the present study with adult zebrafish, as all tested doses promoted a reduction in the locomotor activity of the animals, suggesting a similar pharmacological profile in the species investigated.

### 2.4 Anxiolytic Activity of (+)‐Dihydrocarvone

The experimental protocol was designed in an integrated manner: the open‐field test served as an initial screen for locomotor activity, while the light/dark test, based on the principle of scototaxis, was used as a specific confirmatory test for anxiety, with increased time spent in the light zone serving as a validated parameter for anxiolytic effects. This sequential approach allowed for the discrimination between specific anxiolytic‐like responses and nonspecific motor alterations. Experiments involving pharmacological antagonists provided further mechanistic validation, demonstrating that the observed anxiolytic‐like effects are mediated through specific pathways rather than being a consequence of sedation. This integrated approach is widely used for pharmacological characterization in zebrafish and supports the interpretation of the anxiolytic profile of (+)‐dihydrocarvone [5, 20, 21, 22, 23, 24].

**FIGURE 3:**
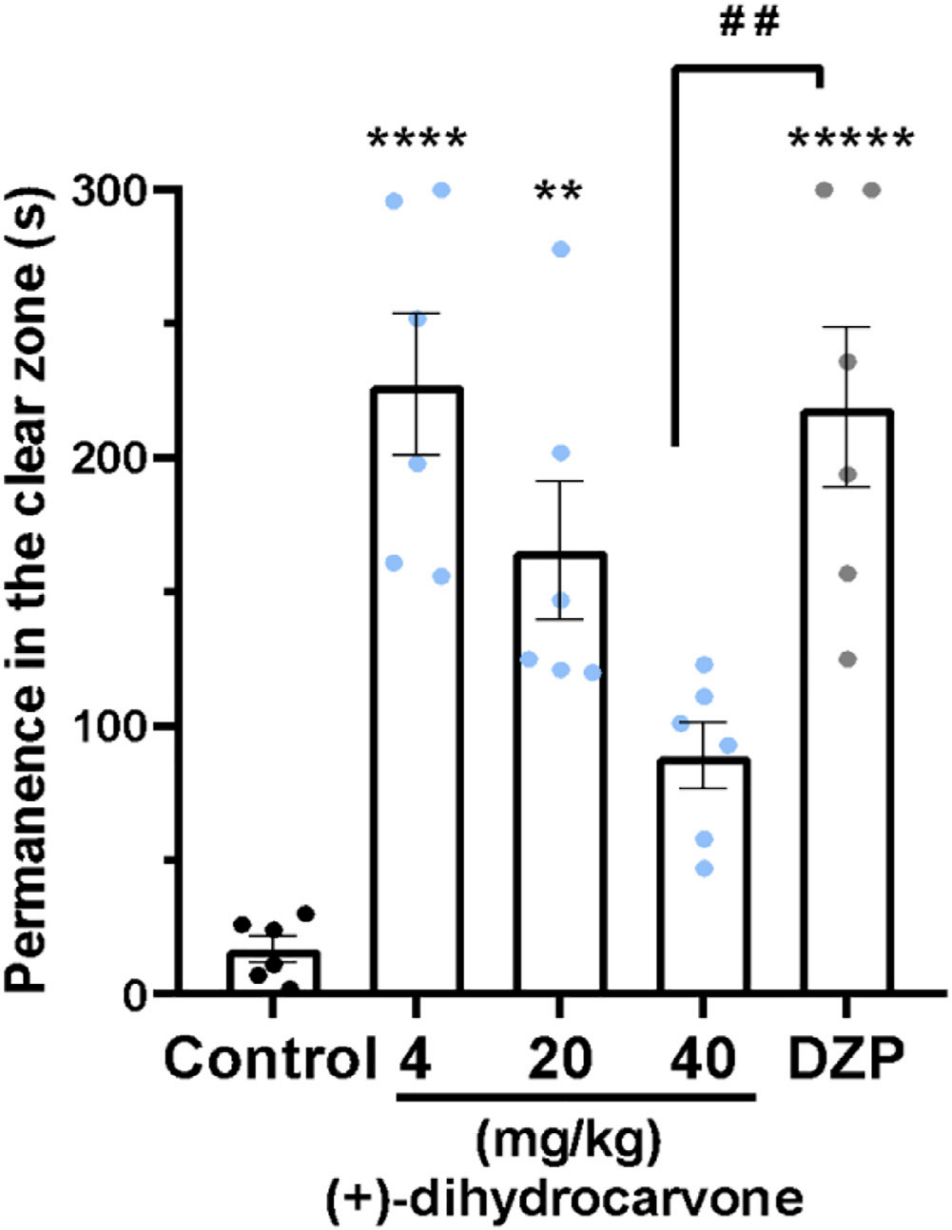
Light/dark test of (+)‐dihydrocarvone in adult zebrafish (*Danio rerio*) (0–5 min). Control (DMSO 3%; 20 µL; *i.p*.); DZP, diazepam (4 mg/kg; *i.p*.). Values represent the mean ± standard error of the mean (SEM) for 6 animals/group; ANOVA followed by Tukey. (**p < 0.01, ****p < 0.0001 vs. control, ##*p* < 0.01 vs. DZP).

Low doses of (+)‐dihydrocarvone (4 and 20 mg/kg) promoted a significant increase (****p < 0.0001 and **p < 0.01 vs. control, respectively) in the time spent by the animals in the light zone of the aquarium. This behavior was analogous to the anxiolytic effect observed in the group treated with DZP, as illustrated in Figure 3. In contrast, the 40 mg/kg dose showed a statistically significant difference compared to the positive control (## *p* < 0.001 vs. DZP) and did not exhibit an anxiolytic effect, suggesting a possible reversal of the response at higher doses.

Previous studies support the pharmacological potential of this class of monoterpenes. Hatano et al. (2012) [25] investigated the effects of repeated treatment with the essential oil of *Lippia alba* (with carvone as the major component) and the enantiomer (R)‐(–)‐carvone in Wistar rats. The authors demonstrated that systemic administration of the essential oil (12.5 and 25 mg/kg, *i.p*.) reduced avoidance latency in the elevated plus maze, indicating an anxiolytic effect similar to that of DZP. The convergence between the results obtained in adult zebrafish in the light/dark test and the data from rodents highlights the promising potential of carvones as anxiolytic agents. These findings are supported by classical literature, which identifies carvone as the major constituent of essential oils traditionally used in medicine for their sedative and tranquilizing properties, acting as a potent depressant of the CNS [26].

### 2.5 Evaluation of GABAergic Neuromodulation—Anxiety

**FIGURE 4:**
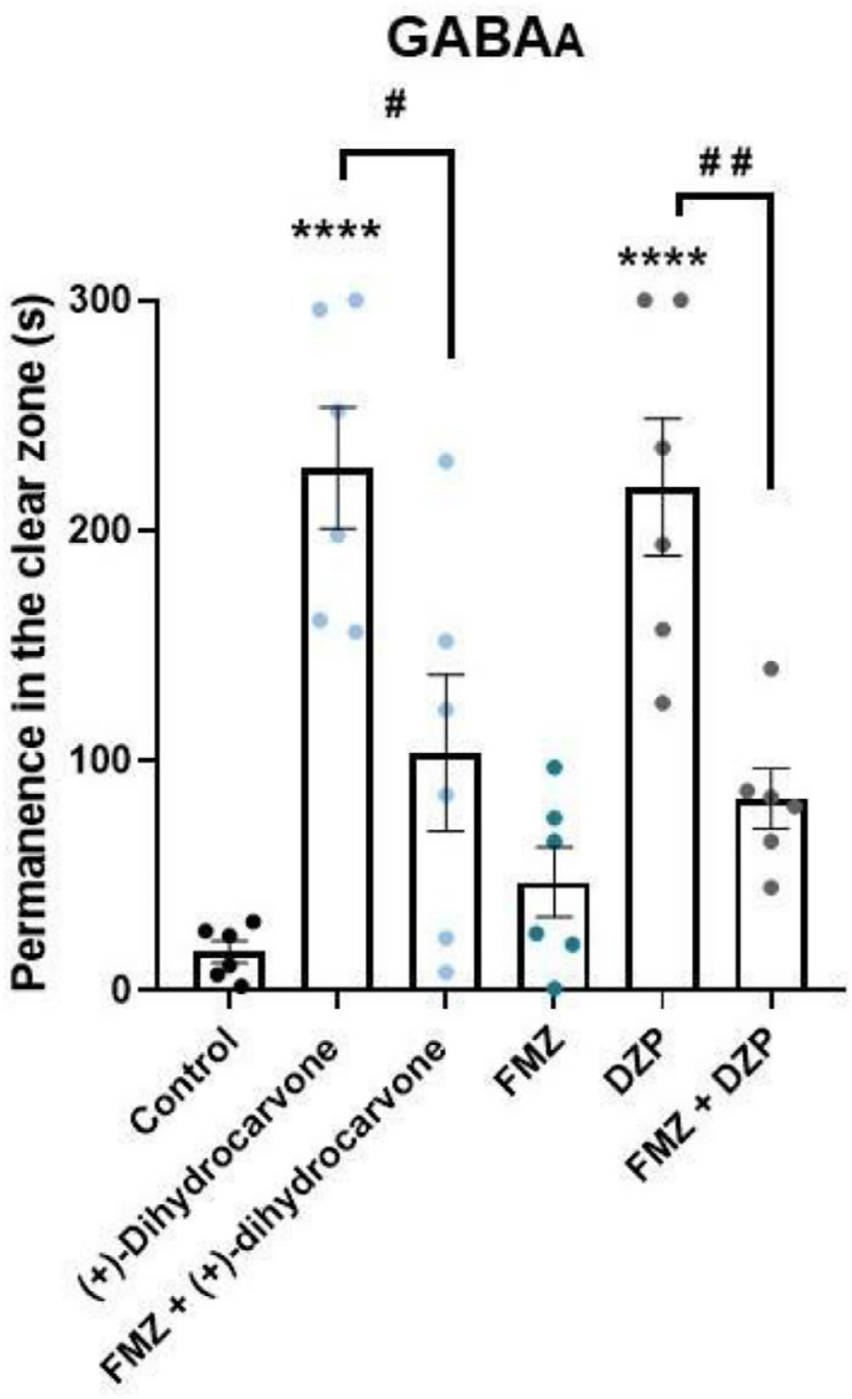
Anxiolytic mechanism of action via GABA of (+)‐dihydrocarvone (4 mg/kg) in adult zebrafish (*Danio rerio*) in the light & dark test (0–5 min). Vehicle—DMSO 3% (20 µL; *i.p*.). DZP—diazepam (4 mg/kg; 20 µL; *i.p*.). Values represent the mean ± standard error of the mean (SEM) for 6 animals/group. Two‐way ANOVA followed by Tukey (**** *p* < 0.0001 vs. Control; #*p* < 0.05 vs. (+)‐dihydrocarvone; ##*p* < 0.01 vs. DZP).

The involvement of GABAergic neurotransmission in the anxiolytic effect of (+)‐dihydrocarvone in adult zebrafish was investigated through pretreatment with flumazenil (a selective antagonist of the benzodiazepine binding site on the GABA_A_ receptor). Flumazenil significantly blocked the effects of (+)‐dihydrocarvone and diazepam (# *p* < 0.05 vs. (+)‐dihydrocarvone; ## *p* < 0.01 vs. DZP), resulting in a drastic reduction in the time spent by the animals in the light zone of the aquarium (Figure 4), which characterizes the reversal of the anxiolytic effect.

Considering the depressant properties of carvone and the mechanisms of action proposed for other monoterpenes [18], de Sousa et al. (2007) [21] suggested that carvone interacts with central GABA_A_ receptors after crossing the blood‐brain barrier. The results in zebrafish corroborate this hypothesis, suggesting that (+)‐dihydrocarvone exerts its anxiolytic activity by interacting with the GABA_A_ receptor complex, possibly at the same binding site as benzodiazepines.

Analyzing the correlation between structure and the anxiolytic activity of (+)‐dihydrocarvone, it is possible to infer that this effect appears to be intrinsically linked to its monoterpene nature. Its small and hydrophobic structure is essential for biological activity, as it allows the molecule to passively cross the blood‐brain barrier. Furthermore, the evidence that flumazenil blocks the effect of (+)‐dihydrocarvone suggests a direct structural correlation with the benzodiazepine allosteric site, where chirality and the presence of the carbonyl functional group facilitate stereospecific interaction with the benzodiazepine binding site on the GABA_A_ receptor. The saturation of the ring, compared to carvone, may confer a spatial conformation that favors positive allosteric coupling, which increases chloride conductance and reduces neuronal excitability.

Anxiolytic and sedative behaviors can appear very similar, particularly with drug classes such as benzodiazepines. Diazepam, used here as a positive control, impaired locomotion and also produced anxiolytic behavior; within this class of drugs, it is difficult to dissociate the anxiolytic effect from the sedative effect. However, it is not possible to determine based solely on the open field test whether reduced locomotion results from sedation. Although open field behavioral tests can identify treatment‐induced changes in locomotion, they cannot determine whether the reduction in movement is due to sedation, muscle relaxation, or anxiolytic behavior. Therefore, the light/dark test was employed to demonstrate the anxiolytic effect, by measuring the increased time the animals spent in the illuminated compartment after treatment. Thus, the results indicate that (+)‐dihydrocarvone produced anxiolytic effects similar to those observed with diazepam, in addition to reducing locomotion. It is possible to deduce that the reduction in locomotion caused by (+)‐dihydrocarvone is not due to sedation, since the doses did not impair memory (see results below), unlike diazepam, which causes motor impairment and also promotes cognitive impairment in the inhibitory avoidance test [14]. Therefore, it is possible that the effects on locomotion produced by diazepam and (+)‐dihydrocarvone are caused by different mechanisms. Future studies will involve specific behavioral tests to better distinguish anxiolytic from sedative effects.

### 2.6 Assessment of Serotonergic Neuromodulation—Anxiety

**FIGURE 5:**
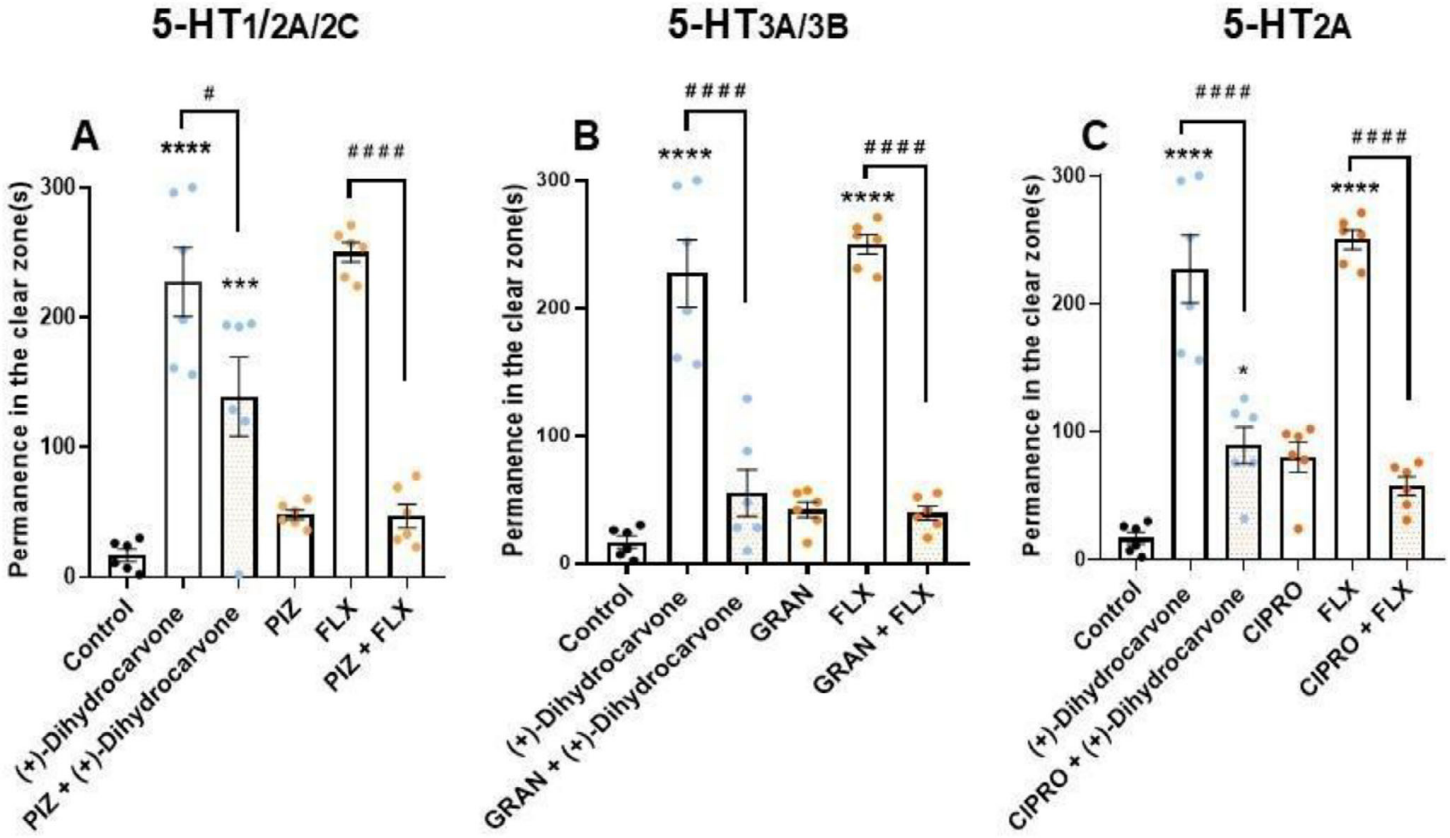
Mechanism of action of anxiolytic action via serotonergic response of (+)‐dihydrocarvone (4 mg/kg) in adult zebrafish (*Danio rerio*) in the light & dark test (0–5 min). Vehicle—DMSO 3% (20 µL; *i.p*.) FLX, fluoxetine (0.05 mg/kg, *i.p*.). Values represent the mean ± standard error of the mean (SEM) for 6 animals/group. Two‐way ANOVA followed by Tukey (*p < 0.05; ***p < 0.001; ****p < 0.0001 vs. control; #*p *< 0.05; ####*p* < 0.0001 vs. FLX).

The evaluation of the mechanism of action of (+)‐dihydrocarvone via serotonergic neuromodulation was also conducted, in this case through pretreatment with Pizotifen (a 5‐HT_1_ and 5‐HT_2A/2C_ antagonist), Cyproheptadine (a 5‐HT_2A_ antagonist), and Granisetron (a 5‐HT_3A/3B_ antagonist). It was observed that the lowest anxiolytic dose (4 mg/kg) of (+)‐dihydrocarvone had its effect significantly blocked (#*p* < 0.05, ####*p* < 0.0001 vs. (+)‐dihydrocarvone) by the serotonergic antagonists, a result similar to that observed with the positive control fluoxetine, which also had its anxiolytic effect significantly blocked (####*p* < 0.0001 vs. FLX) by the antagonists pizotifen, granisetron, and cyproheptadine (Figure 5A–C). These data are consistent with the involvement of serotonergic pathways, suggesting that (+)‐dihydrocarvone may induce anxiolytic behavior through modulatory effects on 5‐HT∼1∼‐, 5‐HT∼2A/2C∼‐, and 5‐HT∼3A/3B∼‐related signaling.

The antidepressant potential of (R)‐(–)‐carvone via serotonergic neuromodulation in mice has already been reported in the literature [19]. The results demonstrated that the effects of carvone were suppressed after pretreatment of the mice with a 5‐HT_1A_ antagonist (WAY‐100,135), a 5‐HT_2A/2C_ antagonist (ketanserin), and a 5‐HT synthesis inhibitor (pCPA). In our study, the anxiolytic behavior induced by (+)‐dihydrocarvone was blocked by pre‐treatment with serotonin receptor antagonists targeting the 5‐HT1 and 5‐HT2A/2C pathways; furthermore, we observed a blockade of this anxiolytic behavior following pretreatment with a 5‐HT3A/3B pathway antagonist. These results suggest that carvone and its derivatives exert effects on the CNS through serotonergic modulation. However, it can be inferred that the anxiolytic action of carvone and its derivative (+)‐dihydrocarvone may involve both GABAergic and serotonergic mechanisms.

### 2.7 Pentylenetetrazole (PTZ)‐Induced Seizure

**FIGURE 6:**
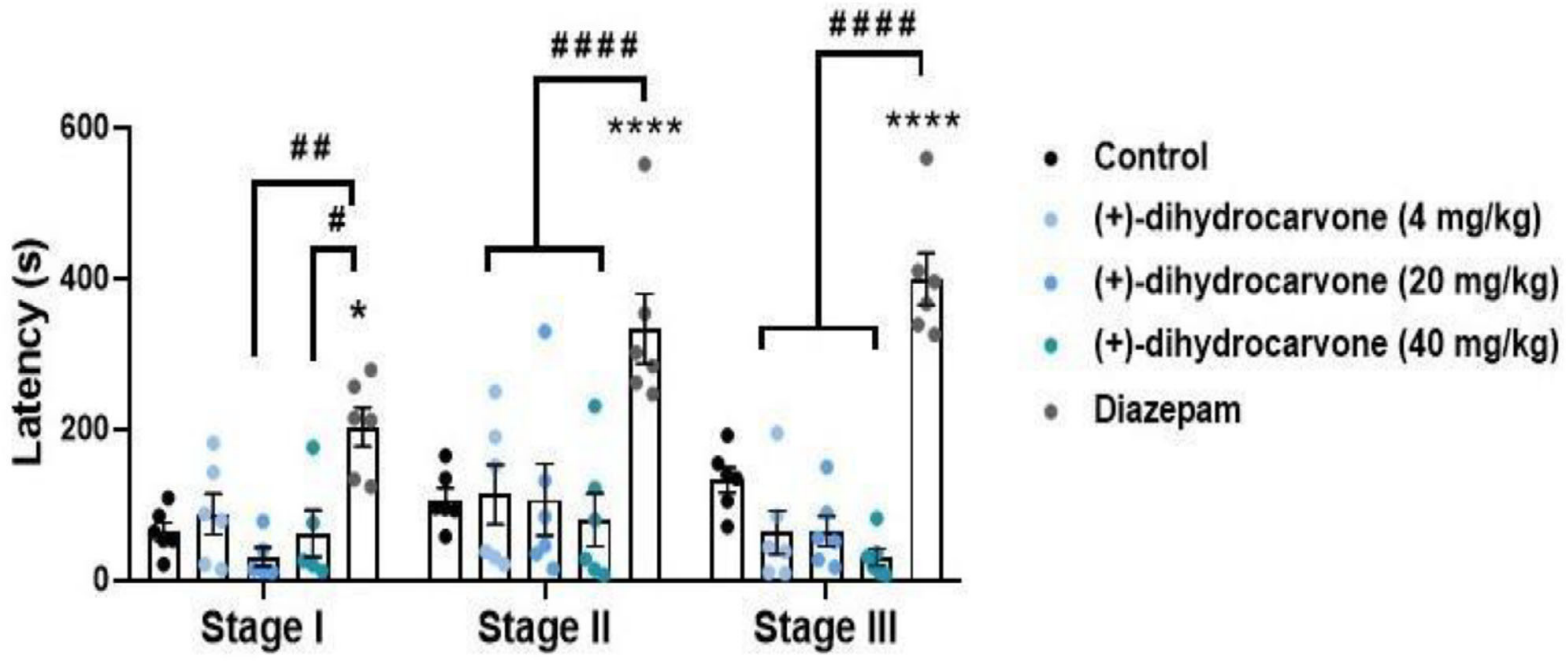
GABA‐mediated anticonvulsant mechanism of (+)‐dihydrocarvone (4, 20, and 40 mg/kg) (A) Stage I. (B) Stage II (C) Stage III. Adult (*Danio rerio*) in the PTZ seizure induction test. Control—DMSO 3% (20 µL; *i.p*.). DZP, diazepam (4 mg/kg; 20 µL; *i.p*.). Values represent the mean ± standard error of the mean (SEM) for 6 animals/group. ANOVA followed by Tukey (*p < 0.05, ***p < 0.01, ****p < 0.0001 vs. control, ##*p* < 0.01 ###*p* < 0.001, ####*p* < 0.0001 sample vs. sample + FMZ or DZP vs. FMZ + DZP).

Unlike what was observed for Diazepam (4 mg/kg), (+)‐dihydrocarvone was not able to reverse the convulsive behavior induced by PTZ at the evaluated doses. The group treated with DZP showed a significant delay in the onset of all three convulsive stages in zebrafish when compared to the control group (3% DMSO) [(*p < 0.05, stage I; ****p < 0.0001, stages II and III)] (Figure 6). These findings indicate that, under the experimental conditions employed, (+)‐dihydrocarvone does not exhibit prominent anticonvulsant activity.

Benzodiazepines are widely used in clinical practice for both the management of seizures and the control of anxiety disorders due to their ability to positively modulate GABA_A_ receptors in the CNS. This interaction enhances neuronal inhibition, reducing brain excitability and raising the seizure threshold [5]. Although the present study demonstrated that (+)‐dihydrocarvone exerts an anxiolytic effect via GABAergic neuromodulation (sensitive to flumazenil), the absence of protection against PTZ suggests a dose‐dependent dissociation or functional selectivity for specific GABA_A_ receptor subtypes. Despite the lack of anticonvulsant efficacy, this negative finding is scientifically valuable, as it clarifies the pharmacological boundaries of (+)‐dihydrocarvone. It demonstrates that the GABAergic modulation responsible for the anxiolytic‐like effect does not generalize to a broad anticonvulsant profile, reinforcing the hypothesis of functional selectivity for distinct GABA_A_ receptor subtypes. This dissociation is particularly relevant from a translational perspective, as it suggests that (+)‐dihydrocarvone may offer anxiety relief without the pronounced sedative, amnestic, and anticonvulsant side effects associated with non‐selective benzodiazepines. Furthermore, this negative result provides critical guidance for future structure–activity relationship studies, helping to delineate the structural features required for anxiolytic efficacy versus those that lead to global CNS depression.

### 2.8 Inhibitory Avoidance

**FIGURE 7:**
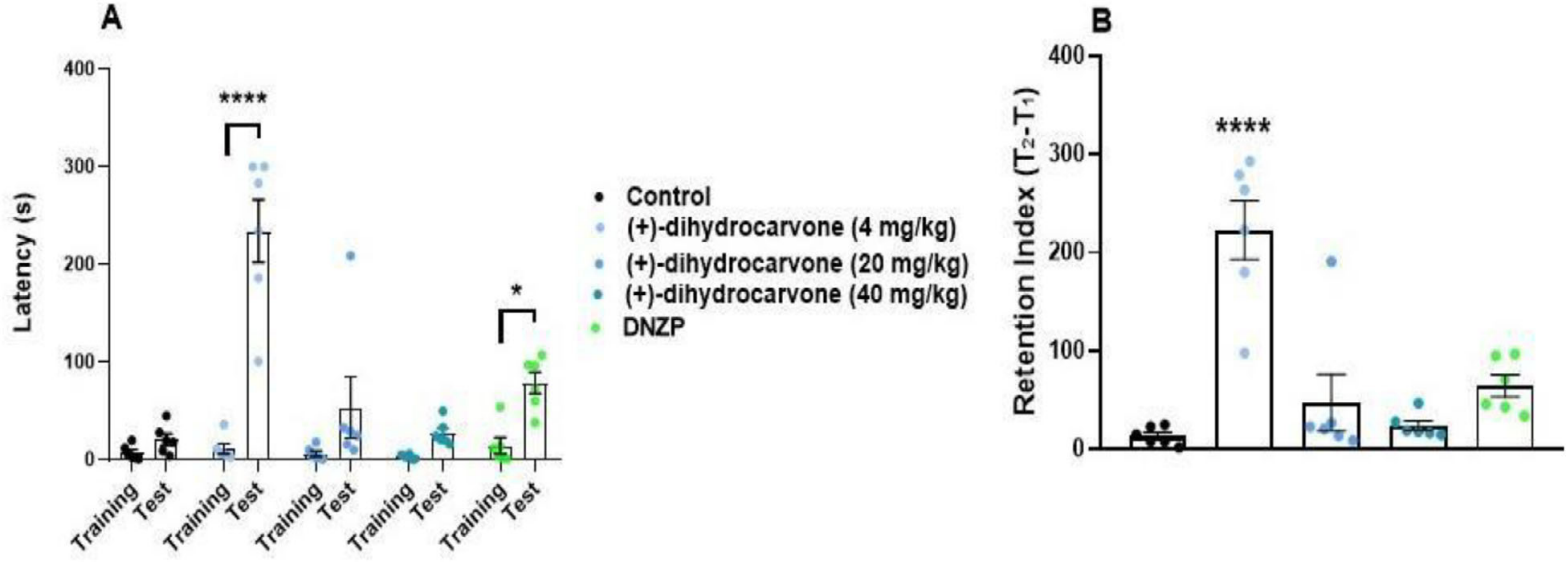
Assessment of (+)‐dihydrocarvone aversive memory in zebrafish studied in the inhibitory avoidance task. (A) Latency to enter the dark area in training and test sessions. (B) Memory retention indices. Values represent the mean ± standard error of the mean for 6 animals/group; Two‐way ANOVA followed by Tukey's test (****p < 0.0001 vs. training). (****p < 0.0001 vs. control) Control‐DMSO (3% dimethyl sulfoxide). DNZP, donepezil (0.5 mg/L).

The results obtained in the inhibitory avoidance test indicated that the administration of (+)‐dihydrocarvone (4 mg/kg) and donepezil (0.5 mg/L) preserved memory consolidation in zebrafish (*p < 0.05 and ****p < 0.0001 vs. training, respectively). This effect was evidenced by a significant increase in the latency to transition to the aversive (dark) compartment during the test session compared to the training session, reflecting successful associative learning (Figure 7A,B). Although benzodiazepines are widely used in clinical practice for their anxiolytic and sedative properties, their use at high doses is often associated with the induction of amnesia [27]. In the present study, it was observed that (+)‐dihydrocarvone produced an anxiolytic effect without compromising cognitive function or memory retention. This finding highlights the therapeutic relevance of carvones, suggesting a promising pharmacological profile for the treatment of anxiety with a reduction in severe adverse effects, such as the mnemonic impairment characteristic of conventional anxiolytics.

### 2.9 Molecular Docking

**FIGURE 8:**
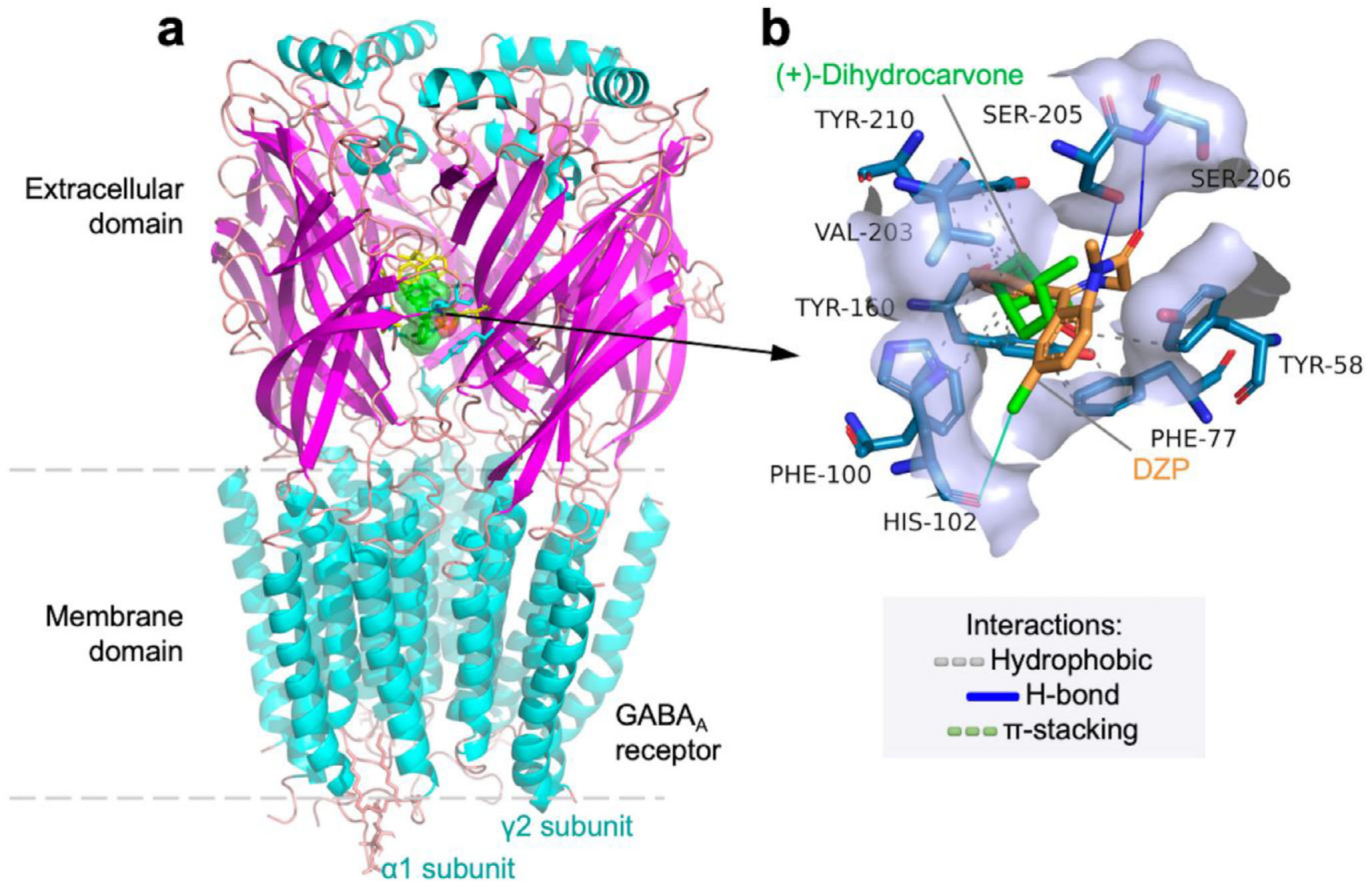
(a) Three‐dimensional (3D) visualization of the GABA_A_ receptor and analysis of ligand–receptor interactions of the ligand (b) (+)‐dihydrocarvone (green) and the agonist diazepam (orange).

In molecular docking simulations, it was observed that (+)‐dihydrocarvone formed a stable complex with the GABA_A_ receptor, presenting an affinity energy of −6.032 kcal/mol, as described in Table 3. It was found that (+)‐dihydrocarvone bound to the same binding site occupied by the reference drug DZP (Figure 8a), which couples the benzodiazepine binding domain (γ2 and α1 subunits) with a root mean square deviation (RMSD) of 1.495 Å of the pose reproduced with redocking (Table 3) [28]. Regarding the interactions involved in the formation of the ligand‐protein complex, the (+)‐dihydrocarvone–GABA_A_ complex predominantly exhibited hydrophobic interactions with seven residues: PHE‐77C, PHE‐100D, TYR‐160D, VAL‐203D, and TYR‐210D (Figure 8b and Table 3). The RMSD was used as a validation criterion for the performed simulations (docking protocol validation), which measures the average distance between the atoms of the superimposed protein‐ligand complexes. It was observed that (+)‐dihydrocarvone showed an RMSD value within the ideal parameter, less than 2 Å, with an RMSD value of 1.956 Å (Table 3).

**TABLE 3:** Molecular docking simulation data expressed in affinity energy, RMSD, and ligand–receptor interactions, expressed in type, residue, and distance.

Receptor	Ligand	Score functions		Interactions	
		Affinity energy (kcal/mol)	RMSD (Å)	Type	Residue and distance (Å)
GABA_A_	(+)‐Dihydrocarvone	−6.032	1.956	Hydrophobic	PHE‐77C; 3.53 PHE‐77C; 3.50 PHE‐100D; 3.60 TYR‐160D; 3.71 VAL‐203D; 3.30 TYR‐210D; 3.60 TYR‐210D; 3.86
	DZP	−6.632	1.495	Hydrophobic	TYR‐58C; 3.52 PHE‐77C; 3.47 PHE‐100D; 3.83 PHE‐100D; 3.92 TYR‐160D; 3.56 VAL‐203D; 3.95 TYR‐210D; 3.38 TYR‐210D; 3.86
				Hydrogen bonds	SER‐205D; 2.94 SER‐206D; 3.16 HIS‐102D; 3.82 SER‐206D
				Halogen Bonds	HIS‐102D

With the results, it is possible to observe that the lipophilic nature of the Fsp3 portion of (+)‐dihydrocarvone favors coupling in the hydrophobic cavity of the agonist located between the γ2 (C‐chain) and α1 (D‐chain) chains of the GABA_A_ receptor, located in the extracellular domain of the protein (Figure 4a) [29]. These results offer important insights that (+)‐dihydrocarvone may exert an anxiolytic effect similar to the DZP agonist, as a possible allosteric modulator of GABA_A_ receptors.

## 3 Conclusions

This is the first study to investigate the neurobehavioral effects of (+)‐dihydrocarvone. The results revealed that this monoterpene showed no acute toxicity in adult zebrafish and induced anxiolytic‐like behavior at the lowest doses tested. Mechanistic analysis demonstrated that (+)‐dihydrocarvone may exert its effects through both GABAergic and serotonergic neurotransmission, while also preserving memory consolidation in adult zebrafish. Molecular docking simulations corroborated the experimental data, suggesting that (+)‐dihydrocarvone interacts with the benzodiazepine binding site of the GABA_A_ receptor predominantly through hydrophobic interactions. In summary, (+)‐dihydrocarvone emerges as a promising candidate for the development of new therapeutic strategies against anxiety, minimizing cognitive adverse effects and establishing a solid foundation for future neuropharmacological investigations.

## 4 Experimental Section

### 4.1 Spectroscopic Characterization of (+)‐Dihydrocarvone in ATR‐FTIR

For the analysis of the (+)‐dihydrocarvone sample, an attenuated reflection Fourier transform infrared spectrometer (ATR‐FTIR) VERTEX 70 V, manufactured by Bruker, under vacuum using the ATR Platinum accessory with diamond crystal.

### 4.2 Drugs and Reagents

The following reagents were used: (+)‐dihydrocarvone (Sigma–Aldrich CAS 5524‐05), diazepam (DZP, Neo Química), flumazenil (FMZ, Sandoz), dimethyl sulfoxide (3% DMSO; Dynamic), pentylenetetrazol (PTZ, Sigma–Aldrich), granisetron hydrochloride, pizotifen maleate, and cyproheptadine. DMSO at 3% was chosen as the vehicle based on its widespread use as an inert control in zebrafish behavioral assays, with no detectable behavioral effects reported at this concentration in adult zebrafish [28, 29].

### 4.3 General Protocol

Adult zebrafish (*D. rerio*) of both sexes, weighing between 0.3 and 0.5 g and aged 90 to 120 days, were used. The animals were housed in glass aquaria (30 × 15 × 20 cm; 10 L) at a maximum density of 10 fish per aquarium, under a 14:10 h light/dark cycle (lights on at 07:00) and a temperature of 27°C ± 1°C, with continuous aeration and submerged filtration; water quality was maintained at pH 7.0 ± 0.2, conductivity of 500–800 µS/cm, ammonia < 0.1 mg/L, and nitrite < 0.1 mg/L. The fish were fed twice daily with commercial flake food (TetraMin) *ad libitum*. The animals were acclimatized to laboratory conditions prior to the experiments and to the testing room before each behavioral assay.

After anesthesia in ice‐cold water, the zebrafish were placed on a damp sponge and treated intraperitoneally (*i.p*.) with 20 µL of the samples (4, 20, or 40 mg/kg), diazepam (4 mg/kg), or 3% DMSO (control); they were then kept individually in 250 mL beakers containing 150 mL of aquarium water.

### 4.4 96‐Hour Acute Toxicity

The Zfa (*n* = 6/group) were treated with the sample at doses of 4, 20, and 40 mg/kg (20 µL). The negative control group received 3% DMSO. Mortality was assessed at 96 h, and the data obtained were statistically analyzed to determine the median lethal dose (LD_50_) using the trimmed Spearman–Karber method, with a 95% confidence interval [31].

### 4.5 Evaluation of Locomotor Activity/Open Field

To evaluate locomotor changes, adult zebrafish (*D. rerio)* (*n* = 6) were treated with the sample (4, 20, and 40 mg/kg, 20 µL), 3% DMSO (negative control), and diazepam (4 mg/kg) (positive control) via intraperitoneal injection. Thirty minutes after application, the animals were placed in Petri dishes (10 × 15 cm) divided into quadrants, and the number of crossings was determined over a period of 5 min [20].

### 4.6 Evaluation of the Anxiolytic Activity of (+)‐Dihydrocarvone

Anxiolytic behavior was evaluated using the light/dark test in glass tanks (30 × 15 × 20 cm) divided into two zones—one light (illuminated at 150–200 lux) and one dark—containing 3 cm of non‐chlorinated water. All behavioral tests were conducted between 9:00 AM and 2:00 PM to minimize circadian variations. Adult zebrafish (*D. rerio*) (*n* = 6) were treated with the sample (4, 20, and 40 mg/kg), 3% DMSO (negative control), and diazepam (4 mg/kg, positive control). After 30 min, the fish were individually transferred to the tank and allowed an acclimatization period; subsequently, the time spent in the light zone was recorded over 5 min [21].

### 4.7 Evaluation of Gabaergic Neuromodulation—Anxiety

To investigate the anxiolytic mechanism, adult zebrafish (*D. rerio*) (*n* = 6) underwent pretreatment with flumazenil (FMZ, 4 mg/kg, *i.p*.), a GABA_A_ antagonist. After 15 min, the effective dose of the sample (4 mg/kg) was applied, as controls we used 3% DMSO and diazepam (4 mg/kg) as negative and positive controls, respectively. Then, the animals were subjected to the light/dark test again as described previously [22].

### 4.8 Assessment of Serotonergic Neuromodulation—Anxiety

To evaluate the anxiolytic mechanism via the serotonergic pathway, adult zebrafish (*Danio rerio*) received a pretreatment with cyproheptadine (5‐HT2A antagonist, 32 mg/kg, *p.o*.), pizotifen (5‐HT1 and 5‐HT2A/2C antagonist, 32 mg/kg, *p.o*.), and granisetron (5‐HT3A/3B antagonist, 20 mg/kg, *p.o*.) [22]. After 30 min, the best effective dose (4 mg/kg) was applied. Flx (20 µL, *i.p*.) and 3% DMSO (20 µL, *i.p*.) were used as controls. The fish were then subjected to the light/dark test again.

### 4.9 Pentylenetetrazole (ptz)‐Induced Convulsion

For seizure inhibition assessment, adult zebrafish (*D. rerio*) (*n* = 6/group) were treated with the sample (4, 20, and 40 mg/kg; 20 µL). Sixty minutes after application, the animals were individually submerged in 7.5 mM PTZ in 250 mL beakers dissolved in distilled water. The three stages observed are classified as: Stage I—dramatic increase in swimming activity; Stage II—whirlpool swimming behavior; Stage III—clonic convulsions, followed by loss of posture when the animal falls to the side and remains immobile for 1‐3 s [23].

### 4.10 Inhibitory Avoidance Test

Inhibitory avoidance was assessed in a glass aquarium (28 × 14.7 × 19 cm; 1.3 L) divided into light and dark compartments, separated by a guillotine door (10 × 10 cm). Adult zebrafish (*n* = 6 per group) were isolated and acclimated for 1 min in the light compartment, and the latency to migrate to the dark compartment was recorded. Upon entering the dark side, the guillotine door was lowered and the aversive stimulus—a mild electric shock (125 mA, 3 ± 0.2 V; 100 Hz) for five seconds—was delivered. After training with the aversive stimulus in the dark compartment, the groups of trained animals received the test compound at the three indicated doses (4, 20, and 40 mg/kg, *i.p*.). In addition, two other groups were treated: one with donepezil (0.5 mg/L) as a positive control, and another with 3% DMSO as a negative control (vehicle for the test compound). After 24 h, the test was repeated without administering the shock, and the latency for the animals to enter the dark compartment (where the aversive stimulus had previously occurred) was measured over a five‐minute period. A longer latency to enter the dark compartment indicated greater memory retention [24].

### 4.11 Molecular Docking

The chemical structure of (+)‐dihydrocarvone was optimized using the steepest descent algorithm, configured in the Avogado.2 software (https://two.avogadro.cc/) to apply the Merck Molecular Force Field (MMFF94) method.

The 3D co‐crystallized structure of the GABA_A_ receptor (PDB ID: 6HUP) was retrieved from the RCSB Protein Data Bank repository (https://www.rcsb.org/), classified as a membrane receptor in Homo sapiens organisms. For protein preparation, water (H_2_O) residues and co‐crystallized ligands were removed, and polar hydrogens were added. Polar hydrogens were added according to the protonation state of the amino acid residues at physiological pH (∼7.4), and then the Gasteiger charges were calculated using AutoDock Tools software (https://autodocksuite.scripps.edu/adt/). Grid‐box parameters were also configured with dimensions *x* = 94 Å, *y* = 92 Å, and *z* = 126 Å, and axes *x* = 134.39, *y* = 135.69, and *z* = 133.776, configured to encompass the entire conformational space of the 3D structure [29].

Autodock Vina version 1.1.2 software was used for molecular docking, which was configured to perform a cycle of 50 independent docking simulations using the multiligand‐based screening model proposed by Eberhardt et al. (2021) [30]. As a validation criterion, redocking was performed using the co‐crystallized ligand DZP and the reference pose located in the extracellular domain of the GABA_A_ receptor, with the repositioning criteria being an RMSD of less than 2.0 Å and an affinity energy of less than −6.0 kcal/mol [32].

### 4.12 Statistical Analysis

Results were expressed as mean values ± standard error of the mean (SEM) for each group of six animals. The sample size (*n* = 6 per group) was based on previous behavioral studies with zebrafish that demonstrated sufficient statistical power to detect significant differences in similar tests [5, 20, 21, 22, 23, 24]. After confirming the normality of the distribution (Shapiro–Wilk test) and the homogeneity of variances (Bartlett's test), differences between groups were analyzed using one‐way or two‐way ANOVA, followed by Tukey's post hoc test. All analyses were performed using GraphPad Prism v. 8.0 software. The level of statistical significance was set at 5% (*p* < 0.05).

Behavioral assessments were conducted by a blinded researcher who was unaware of the treatment conditions. Animals exhibiting abnormal swimming, visible injuries, or extreme stress prior to treatment were not included in the experiments.

## Author Contributions

**Isadora Freitas de Sousa**: formal analysis, and writing – review and editing; **Francisco Rogênio da Silva Mendes**: investigation, formal analysis, and writing – review and editing; **Antônio Wlisses da Silva** and **Maria Kuerislene Amâncio Ferreira**: formal analysis, software, validation, and writing – review and editing; **Leticia Souza da Silva** and **Matheus Nunes da Rocha**: methodology, formal analysis, and writing – review and editing; **Jane Eire Silva Alencar de Menezes**, **Emmanuel Silva Marinho**, and **Márcia Machado Marinho**: conceptualization and writing – review and editing; **Hélcio Silva dos Santos**, **Antônio César Honorato Barreto**, and **Alexandre Magno Rodrigues Texeira**: funding, supervision and writing – review and editing.

## Conflicts of Interest

The authors declare no conflicts of interest.

## Data Availability

The data that support the findings of this study are available from the corresponding author upon reasonable request.
